# Rare cause of repeated pulmonary embolism: a case of primary pleural squamous cell carcinoma and literature review

**DOI:** 10.1186/s12890-020-1077-2

**Published:** 2020-03-26

**Authors:** Zhongzhong Chen, Tingting Feng, Meng Wang, Xingxiang Xu, Yuxiu Wang, Yiran Li, Lingfeng Min

**Affiliations:** Department of Respiratory and Critical Care Medicine, Northern Jiangsu People’s Hospital, Dalian Medical University, Clinical Medical College of Yangzhou University, Yangzhou, 225001 Jiangsu China

**Keywords:** Pulmonary embolism, Primary pleural squamous cell carcinoma, Pleural malignancy

## Abstract

**Background:**

Malignant tumors are risk factors for a pulmonary embolism (PE), and a PE caused by a tumor is not uncommon. Primary pleural squamous cell carcinoma (PPSCC) is a rare malignancy; thus, a related PE is extremely rare.

**Case presentation:**

A previously healthy 49-year-old female patient was admitted to Northern Jiangsu People’s Hospital owing to chest tightness, cough, and breathing difficulty that persisted for 3 days. Following admission, a computed tomography (CT) pulmonary angiography revealed an embolism in the main pulmonary artery, upper and lower pulmonary artery branch. The patient was treated with alteplase, warfarin, and antibiotics. Over the following year, she experienced recurrent chest pain and tightness and breathing difficulty, with multiple CT pulmonary angiography revealing thrombosis in the right and left main pulmonary artery. No abnormalities were observed in surrogate markers of autoimmune diseases, tumor antigen testing, or ultrasonography; thus, the cause of recurrent PE was not identified. Subsequently, a positron emission tomography-computed tomography (PET-CT) examination revealed diffuse heterogeneous thickening of the right pleura and substantially increased glucose metabolism. A CT-guided pleural biopsy was performed, and histopathological examination of the pleura eventually revealed a diagnosis of PPSCC.

**Conclusions:**

PPSCC is a rare tumor that lacks specific clinical manifestations and is difficult to detect with imaging techniques. The occurrence of PE as the primary manifesting symptom in a patient with PPSCC is extremely rare. Thus, malignant tumors should be considered in patients with no risk factors for PE and/or in those with recurrent PE. An immediate diagnosis and adequate intervention can be achieved with increased awareness of this diagnosis and subsequent related examinations.

## Background

Primary tumors of the pleura are uncommon and primary pleural squamous cell carcinoma (PPSCC) is an exceedingly rare pleural malignancy which has seldom been reported [1, 2]. The clinical manifestations of PPSCC are not typical. In the early stages, patients with PPSCC are usually asymptomatic, with localized pleural thickening or small nodules noted on computed tomography (CT) [3], which are difficult to detect, often leading to a misdiagnosis [2].

Venous thromboembolic events comprise deep vein thrombosis and pulmonary embolism (PE), which are common causes of morbidity and mortality, particularly in patients who are hospitalized or bedridden [4]. Malignancy is a known risk factor for venous thromboembolism; venous thromboembolism caused by malignant tumors is clinically not rare [5]. However, the onset of PPSCC characterized by a PE is extremely rare and has not been reported in the literature to date. In the present study, we described a case of PPSCC with PE as the first manifestation. Because of this unusual presentation, it was difficult to clinically detect the tumor, resulting in delayed accurate diagnosis and appropriate treatment.

## Case presentation

**Fig. 1:**
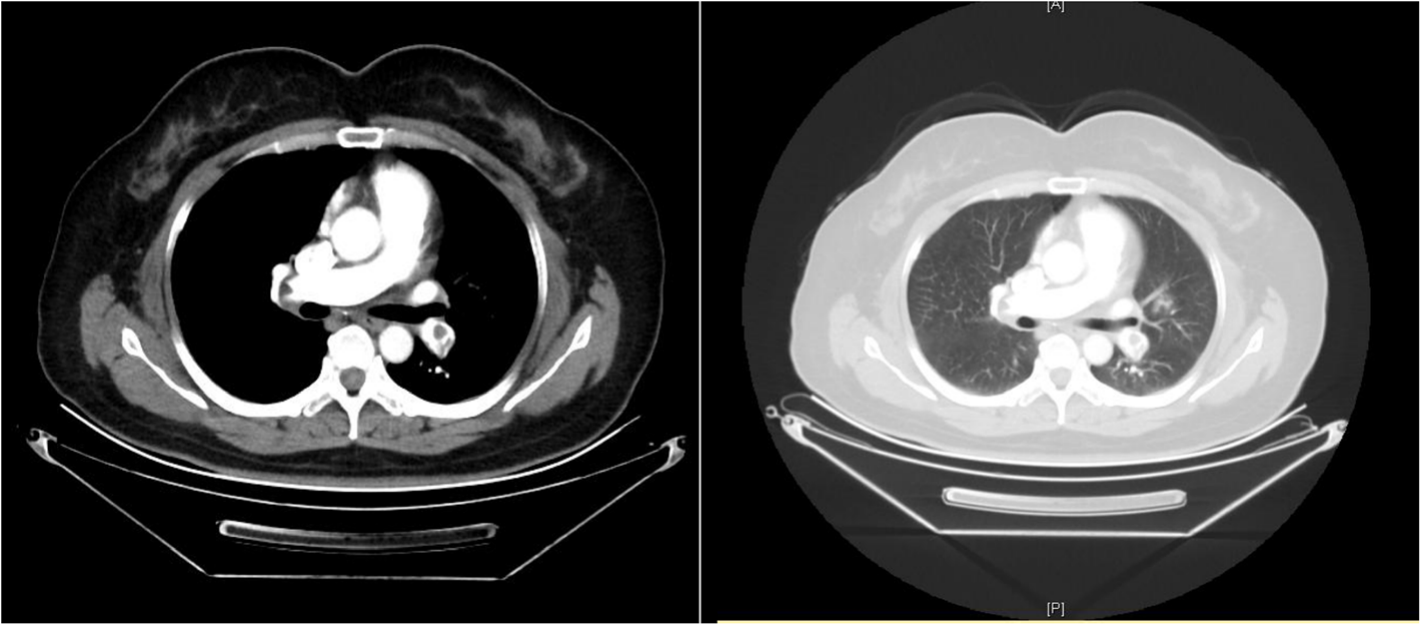
Computed tomography pulmonary angiography (CTPA) revealing filling defects in the main pulmonary artery and upper and lower pulmonary artery branch

In June 2017, a 49-year-old Chinese woman was admitted to the emergency department of the Northern Jiangsu People’s Hospital owing to chest tightness and breathing difficulty that persisted for 3 days. She was previously in good health and was a non-smoker. In addition, she had fever, chills, and a maximum body temperature of 38 °C. She did not have urinary or fecal incontinence, chest pain, general fatigue, cough, or hemoptysis during the course of her disease. Following admission, results of complete blood test revealed that white blood cell count was 12.21 × 10^9^ cells/L and the percentage of large white blood cells was 85.5%. Blood gas analysis results showed 7.480 PH, 100 mmHg PaO_2_, 31 mmHg PaCO_2_, and 23.1 mmol/L HCO^3−^. D-dimer assay indicated a value of 5.28 mg/L. A computed tomography pulmonary angiography (CTPA) revealed filling defects in the main pulmonary artery, upper and lower pulmonary artery branch (Fig. 1). There were no distinct signs of embolism in color ultrasonography examination of the upper and lower limbs. The patient was diagnosed with PE, and was treated with anticoagulation, antiplatelet aggregation, and anti-infection medications. During the 18 h after admission, the patient experienced worsening shortness of breath and anoxia and was subsequently transferred to the Emergency Intensive Care Unit (EICU) for non-invasive ventilator assisted ventilation. Additionally, the patient was intravenously administered alteplase once every 12 h for 3 consecutive days and 5000 units subcutaneous unfractionated heparin. Warfarin (5 mg) was orally administered once a day. On noting that the prothrombin time (PT) and international normalized ratio (INR) were 2–2.5 times their respective normal levels, warfarin therapy was singly administered along with anti-infective, supportive, and oxygen therapies. Once her condition had considerably improved and the anoxia was reduced, she was discharged from the hospital.

**Fig. 2:**
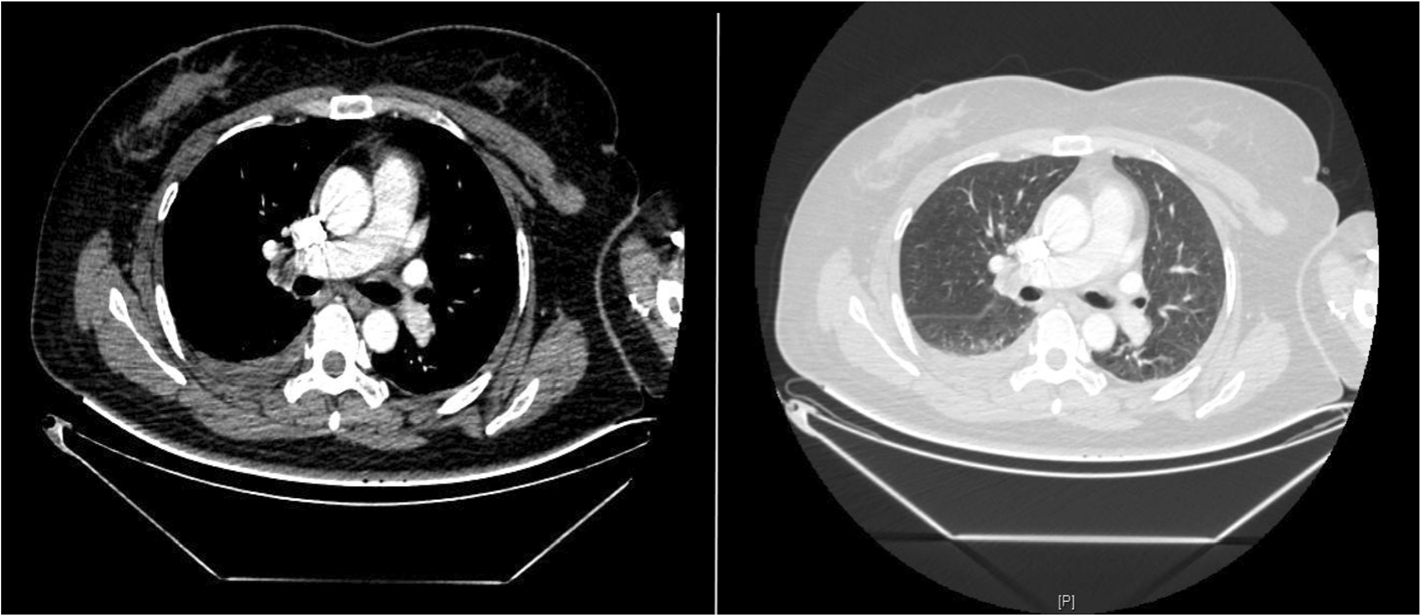
Computed tomography pulmonary angiography (CTPA) revealing filling defects in the left and right branches of pulmonary artery as well as bilateral pleural effusion

After 10 days, dyspnea and chest tightness recurred. In addition, she experienced persistent right chest pain, which aggravated when she lied on her right side and was not relieved by rest. Minor vaginal bleeding was also observed. She presented to the Department of Respiratory and Critical Care Medicine of our hospital for a CTPA, which revealed filling defects in the left and right branches of pulmonary artery as well as bilateral pleural effusion (Fig. 2). A routine coagulation test revealed 37 s PT and 3.33 INR, and a D-dimer assay showed a value of 8.75 mg/L. No abnormalities were observed in limb color ultrasonography, tumor antigen testing, or surrogate markers of autoimmune diseases (anti-nuclear antibody, anti-RNP antibody, anti-CCP antibody, c-ANCA, p-ANCA, anti-SS-A antibody, and anti-SS-B antibody). Based on the monitoring of PT and INR, we decided to discontinue warfarin to prevent an overdose situation. Relevant contraindications were excluded and the patient underwent interventional inferior vena cava filter implantation combined with thrombolysis implantation therapy, along with low-dose urokinase. Following surgery, she continued to receive anticoagulant treatment. On considerably improvement in her condition, she was discharged.

**Fig. 3:**
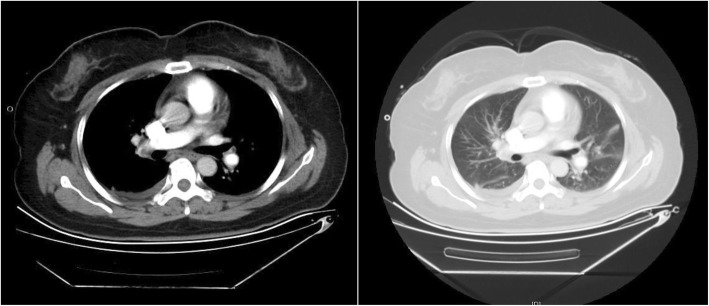
Computed tomography pulmonary angiography (CTPA) revealing no evident improvement of the thrombosis in the right and left main pulmonary artery, but a disappearance of the right pleural effusion

One month later, she was referred to our hospital again due to swelling in her right lower limb. She received interventional inferior right iliac vein dilation and stent placement. In October 2018, she presented again with a week-long history of right-sided chest pain, cough, difficulty in breathing, and weight loss. A CTPA (Fig. 3) revealed no evident improvement of the thrombosis in the right and left main pulmonary artery, progression of pneumonia, and a reduced right pleural effusion, compared with the previous CTPA. Once again, the tumor associated antigen test was performed. Tumor antigen testing revealed an elevated Ca125 level of 69.16 U/ml (normal value < 35 U/ml) and Ca199 level of 30.22 U/ml (normal value < 27 U/ml). She was treated with anticoagulation, anti-infection, supportive, and oxygen therapies. We recommended her to undergo a positron emission tomography-computed tomography (PET-CT) test, but she refused and was discharged from the hospital.

**Fig. 4:**
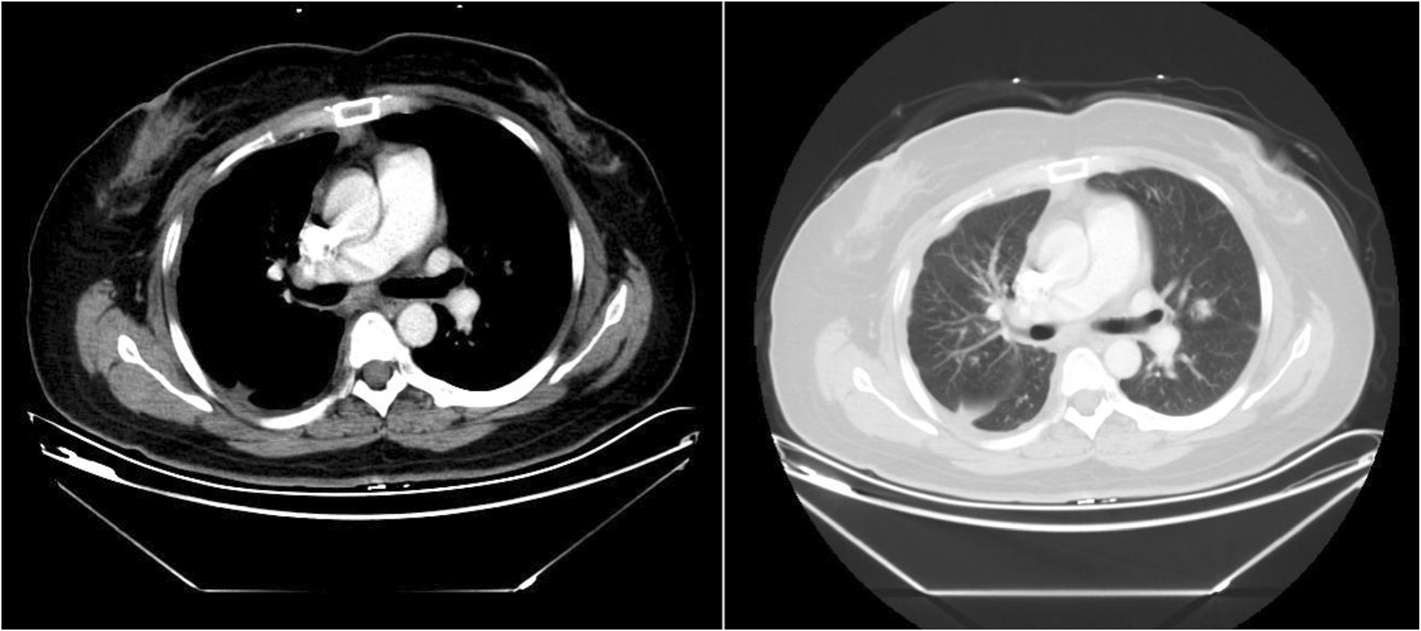
Enhanced chest computed tomography (CT) scan revealing bilateral pulmonary embolism, bilateral pulmonary infection, right pleural thickening and pleural effusion

**Fig. 5:**
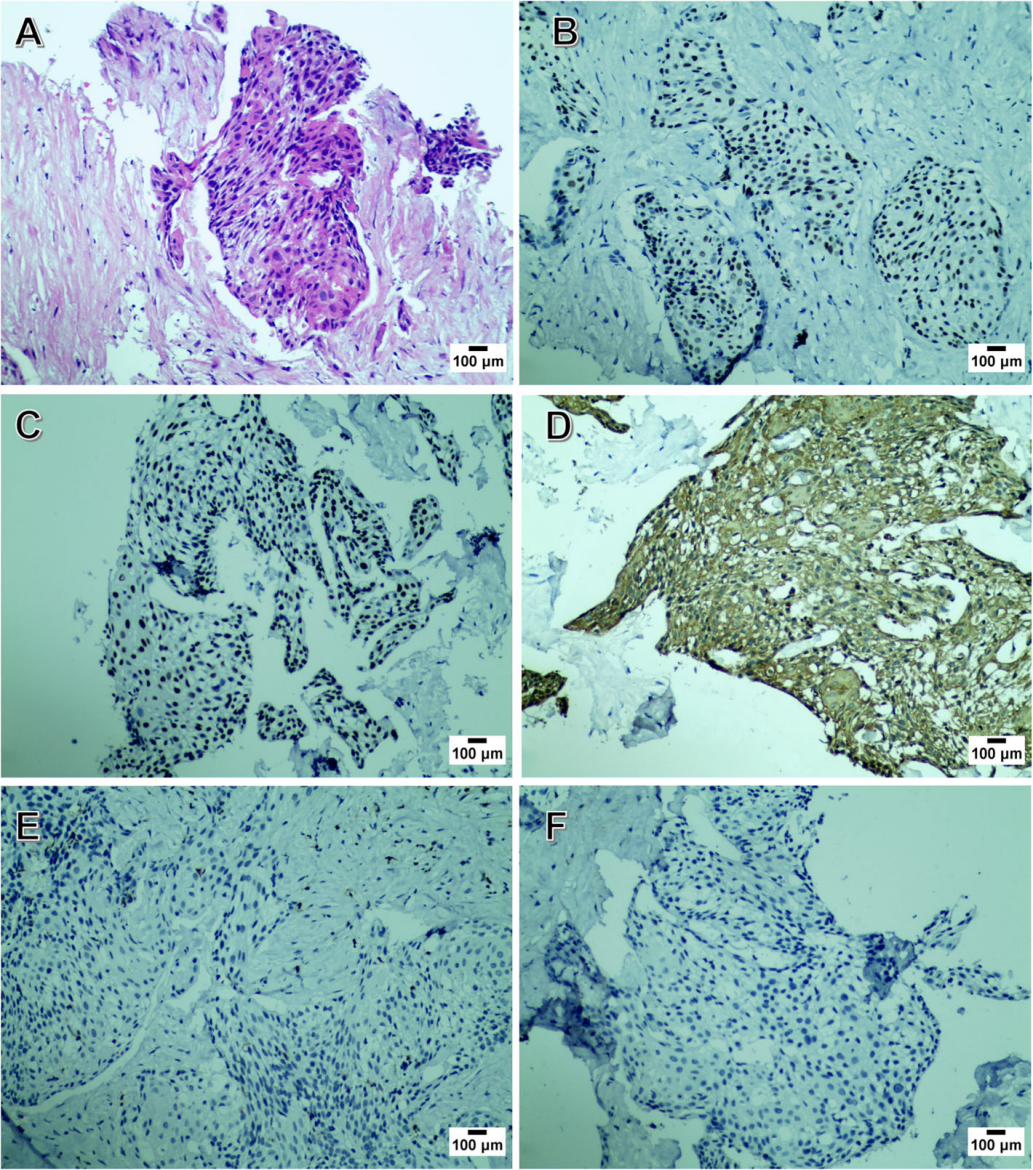
**a** Tumor consisted of squamous cells arranged in the nest bulk with invasive growth (hematoxylin and eosin staining, × 200). **b** IHC staining of P63 was positive, × 200. **c** IHC staining of P40 was positive, × 200. **d** IHC staining of CK5/6 was positive, × 200. **e** IHC staining of CD5 was negative, × 200. **f** IHC staining of Calretinin was negative, ×200

She subsequently underwent a PET-CT examination in another hospital. The PET-CT revealed diffuse heterogeneous thickening of the right pleura, substantially increased glucose metabolism, fluorodeoxyglucose (FDG) uptake on L5 vertebral body, striated soft tissue lesions along the right iliac vein, and increased FDG uptake. In November 2018, she was once again admitted to our hospital. Results of enhanced chest CT scan revealed a bilateral PE, bilateral pulmonary infection, right pleural thickening, and pleural effusion (Fig. 4). She completed the CT-guided pleural biopsy and postoperative histopathology revealed the tumor consisting of squamous cells were arranged in the nest bulk with invasive growth (Fig. 5a). Immunohistochemical (IHC) staining was performed for pleural lesion was positive for P63 (Fig. 5b), P40 (Fig. 5c), CK5/6 (Fig. 5d), epithelial membrane antigen (EMA) and negative for CD5 (Fig. 5e), CD117 and Calretinin (Fig. 5f). Combining with the IHC analysis, the pathological diagnosis was squamous cell carcinoma. Based on her medical history and results of histopathological examination and imaging, the patient was finally diagnosed with PPSCC.

Between 1977 and 2019, we identified 9 previous reports of squamous cell carcinoma arising from the pleura in patients. As described in Table 1, patients ranged in age between 49 and 75. These cases underwent chest pain, cough, dyspnoea, and other clinical symptoms. Most lesions of these patients were observed in the right pleura. Most of the cases had been in long standing chronic empyema, bronchopleural fistula or pneumothorax before a diagnosis of squamous cell carcinoma. But in our case, the patient was previously healthy without chronic empyema or bronchopleural fistula. After being diagnosed with pulmonary embolism, our patient experienced recurrent chest wall pain and pulmonary embolism.

**Table 1:** Clinical features of cases that describe primary pleural squamous cell carcinoma

Case	Age / Sex	Tumor location	Past medical history	Clinical symptoms	Treatment	Follow-up and outcome
Rüttner 1977 [6]- Case 1	49/M	Right	Empyema	NA	S	36 months, Live
Rüttner 1977 [6]- Case 2	43/F	Left	Pneumothorax	Cough, Hemoptysis	S	NA
Garty 1987 [1]	61/M	Right	Empyema	Cough, Chest pain	S	NA
Prabhakar 1989 [7]	58/F	Left	BPF, Empyema	Cough, Hemoptysis	S	5 months, Die
Sapino 1996 [3]	65/M	Right	Pneumothorax	Dyspnea, Chest pain	PT	1 months, Die
Zapatero 2004 [8]	45/M	Right	BPF, Empyema	Fever, Dyspnea	S	6 months, Die
Mark 2010 [9]	67/M	Right	BPF	Chest pain	PT	4 months, Die
Lin 2013 [2]	75/F	Right	NA	Cough, Chest pain	S + R	38 months, Live
Ronchi 2018 [10]	56/M	Right	NA	Cough, Dyspnoea	C	NA
Jeon 2017 [11]- Case 1	74/M	Left	BPF, Empyema	Chest pain	R	NA
Jeon 2017 [11]- Case 2	53/F	Right	Empyema	Chest pain	R	5 months, Die
Present case	49/F	Right	NA	Chest pain	PT	NA

*M* male, *F* female, *BPF* bronchopleural fistula, *S* surgery, *C* chemotherapy, *R* radiotherapy, *PT* palliative treatment, *NA* not available

## Discussion and conclusions

PE is a clinical syndrome in which an artery in the lungs is blocked by a substance such as a gas or thromboembolism, with a thromboembolism being the most common cause. Cancer is a well-known risk factor for PE, and lung cancer is the most common malignancy coexisting in patients with PE [12]. Studies have demonstrated that the risk of thromboembolic events in patients with cancer is much greater than that in the general population [13], with some studies reporting that patients with cancer are at a 4–7-fold higher risk of PE than those without cancer [5, 14]. Blom et al. reported that patients with hematologic malignancies are at the highest risk of PE, followed by those with lung and gastrointestinal cancers [5]. In addition, venous thrombosis is probably a symptom of occult malignancy, with PE often being the first symptom in patients with malignant tumor [15]. Similarly, this patient with PPSCC presented in a subtle manner, with PE as the initial symptom. Owing to the rarity of PPSCC, no published reports exist that describe PPSCC presenting with PE.

The most common pleural malignancies are metastases from primary tumors such as lung or breast cancers [16]. Primary tumors of the pleura are uncommon, accounting for 10% of all pleural neoplasms [17]. Because PPSCC is particularly rare, the pathogenesis and incidence of this tumor is unclear [10]. To date, no association has been demonstrated between PPSCC and environmental factors classically associated with other pleural and pulmonary neoplasms, such as tobacco and asbestos exposure [7]. However, we conducted a literature search and found some cases of squamous cell carcinoma arising from the pleura in patients with chronic empyema or subjected to therapeutic pneumothorax for active tuberculosis [3, 18]. Thus, we can not exclude that chronic inflammation may be one of the causes of PPSCC.

Early PPSCC is usually asymptomatic and morphologically similar to pleural mesothelioma, which is easily misdiagnosed as localized pleural mesothelioma [2]. However, as the disease progresses, patients may experience chest pain, cough, expectoration, fatigue, weight loss, and other atypical symptoms [1, 8]. In our case, the patient was previously healthy without any chronic pleural inflammation. After being diagnosed with PE, our patient experienced right chest pain, dyspnea and hemoptysis. The patient’s pain continued to worsen and was accompanied with pleural effusion. We considered that the local pleural tumor gradually invaded the chest wall, ribs, and intercostal nerves, resulting in bone destruction and continuous pain caused by nerve stretch.

The morphology of PPSCC lacks specificity, and only local pleural thickening can be observed on chest radiography or CT, which cannot distinguish between benign and malignant tumors. Pleural fluid cytology for malignant cells is the simplest way to diagnose malignant tumors, but the diagnostic yield of pleural fluid cytology is low [19]. In recent years, PET-CT has been increasingly used to diagnose and stage primary malignancies [20]. Some reports have confirmed that a PET-CT can accurately distinguish between benign and malignant tumors, as pleural malignancy shows increased FDG uptake [20, 21]. The gold standard for the diagnosis of a pleural tumor involves the pathological analysis of a pleural biopsy. Percutaneous CT-guided Tru-Cut needle biopsies are considered a superior diagnostic method. The reported rate of diagnostic sensitivity on CT guidance to target areas of pleural disease has been shown to increase the sensitivity to 87.5% [22]. However, thoracoscopy pleural biopsy is now widely considered the gold standard diagnostic modality with a diagnostic yield of up to 99% [23, 24].

Early detection, early resection, and thorough removal of tumors are important for the treatment of primary pleura tumors and prevention of recurrence. Total surgical resection is the best treatment for early PPSCC. As a minimally invasive procedure, video-assisted thoracoscopic surgery (VATS) is the optimal method for the removal of early pleural tumors [25, 26]. Early PPSCC can be removed by VATS; if it is not possible to obtain the surgical margin of the tumor using VATS, the procedure should be converted to an open thoracotomy [27]. For tumors with unclear boundaries and suspected malignant pleural nodules, intraoperative frozen examination should be used to determine whether an extended resection should be performed to reduce the risk of recurrence and improve the prognosis [2, 25]. Radiotherapy and chemotherapy are considered for patients with recurrence or for those who are inoperable. However, little data exists regarding the efficacy of radiotherapy and chemotherapy in these patient groups. Owing to the recurrence of PE in our patient, her condition was severe and she was administered palliative treatment.

In conclusion, PPSCC is a rare pleural tumor, with a lack of specificity in its clinical manifestations and related findings on imaging. Early PPSCC is difficult to detect, which may result in substantial delays in the diagnosis and treatment. However, PET-CT is a crucial functional imaging technique for detecting malignant pleural lesions and assessing the extent of tumor involvement. Malignant tumors should be considered in patients with no risk factors for PE and in those with recurrent or refractory PE. PE is likely to be a symptom of occult malignancy. In this situation, a careful physical examination together with imaging-assisted techniques and measurement of tumor markers is of utmost importance to promptly identify a tumor.

## Data Availability

The datasets used and/or analyzed during the current study are available from the corresponding author on reasonable request.

## Abbreviations

CT: Computed tomography
CTPA: Computed tomography pulmonary angiography
EMA: Epithelial membrane antigen
FDG: Fluorodeoxyglucose
IHC: Immunohistochemical
INR: International normalized ratio
PE: Pulmonary embolism
PET-CT: Positron emission tomography-computed tomography
PPSCC: Primary pleural squamous cell carcinoma
PT: Prothrombin time
VATS: Video-assisted Thoracoscopic Surgery
